# A Complex Regulatory Network Coordinating Cell Cycles During *C. elegans* Development Is Revealed by a Genome-Wide RNAi Screen

**DOI:** 10.1534/g3.114.010546

**Published:** 2014-02-28

**Authors:** Sarah H. Roy, David V. Tobin, Nadin Memar, Eleanor Beltz, Jenna Holmen, Joseph E. Clayton, Daniel J. Chiu, Laura D. Young, Travis H. Green, Isabella Lubin, Yuying Liu, Barbara Conradt, R. Mako Saito

**Affiliations:** * Department of Genetics, Geisel School of Medicine at Dartmouth, Hanover, New Hampshire 03755; † Center for Integrated Protein Science Munich (CiPSM), Biocenter, LMU Munich, 82152 Planegg-Martinsried, Germany; ‡ Norris Cotton Cancer Center, Lebanon, New Hampshire 03756

**Keywords:** *C. elegans*, cell cycle, regulatory network, ubiquitin-conjugating enzyme, development, intestine

## Abstract

The development and homeostasis of multicellular animals requires precise coordination of cell division and differentiation. We performed a genome-wide RNA interference screen in *Caenorhabditis elegans* to reveal the components of a regulatory network that promotes developmentally programmed cell-cycle quiescence. The 107 identified genes are predicted to constitute regulatory networks that are conserved among higher animals because almost half of the genes are represented by clear human orthologs. Using a series of mutant backgrounds to assess their genetic activities, the RNA interference clones displaying similar properties were clustered to establish potential regulatory relationships within the network. This approach uncovered four distinct genetic pathways controlling cell-cycle entry during intestinal organogenesis. The enhanced phenotypes observed for animals carrying compound mutations attest to the collaboration between distinct mechanisms to ensure strict developmental regulation of cell cycles. Moreover, we characterized *ubc-25*, a gene encoding an E2 ubiquitin-conjugating enzyme whose human ortholog, UBE2Q2, is deregulated in several cancers. Our genetic analyses suggested that *ubc-25* acts in a linear pathway with *cul-1*/Cul1, in parallel to pathways employing *cki-1*/p27 and *lin-35*/pRb to promote cell-cycle quiescence. Further investigation of the potential regulatory mechanism demonstrated that *ubc-25* activity negatively regulates CYE-1/cyclin E protein abundance *in vivo*. Together, our results show that the *ubc-25*-mediated pathway acts within a complex network that integrates the actions of multiple molecular mechanisms to control cell cycles during development.

The somatic development of the nematode *Caenorhabditis elegans* proceeds through a highly reproducible cell lineage (Sulston and Horvitz 1977; Kimble and Hirsh 1979; Sulston *et al.* 1983). The virtually invariant spatiotemporal cell division pattern can be experimentally exploited to detect subtle defects in the stringent control of cell divisions that result in ectopic cell production (van den Heuvel 2005; Kirienko *et al.* 2010). Several tissues are particularly well suited for studies of developmental regulation of cell cycles. The organogenesis of the nonessential vulva is among the most studied developmental processes of *C. elegans*. The organ can be generated from six vulval precursor cells (VPCs) that arise during the first larval stage (L1) and immediately exit the cell cycle. This period of cell-cycle quiescence ends in the third larval stage (L3), when the cells divide and differentiate into either vulva or hypodermis (skin). The intestine and hypodermis are also of great interest for cell-cycle studies because of the developmentally controlled switch to specialized cell cycles (van den Heuvel 2005). Because the loss of cell-cycle control is a hallmark of cancer (Hanahan and Weinberg 2011), studies of normal cell-cycle regulation during the highly coordinated development of *C. elegans* provides a finely tuned model to study pathways that potentially function in humans.

The mechanisms controlling cell-cycle progression are highly conserved throughout eukaryotes. The orchestrated activation and inactivation of complexes consisting of cyclin-dependent kinases (CDK) and their cyclin partners ensures the orderly progression through the phases of the cell cycle (Nigg 1995; Morgan 1997). The regulation of cyclin/CDK activity is accomplished through the collaboration of several distinct mechanisms, including both transcriptional and post-transcriptional regulation of cyclin expression, post-translational modification of CDK and interaction with CDK inhibitors (Muller 1995; Sherr and Roberts 1999; Obaya and Sedivy 2002; Stevaux and Dyson 2002; Kitagawa *et al.* 2009; Mocciaro and Rape 2012). The normal regulation of the cyclin/CDK complexes controlling the G_1_/S transition frequently are disrupted in human cancers (Sherr 1996). Similarly in *C. elegans*, extra cell division defects can result from dysregulation of the cyclin/CDK complexes controlling G_1_/S progression (van den Heuvel 2005; Kirienko *et al.* 2010); thus, our studies have focused on the mechanisms regulating the activities of G_1_ phase CDK complexes.

To identify the genes acting within a regulatory network that coordinates cell-cycle progression with development, we conducted a genome-wide, reverse genetic screen. Herein we report the 107 genes identified by the screen whose activities were required to establish or maintain an extended period of cell-cycle quiescence during vulva development. Further genetic analyses of the genes suggested that at least four pathways act in parallel to restrict cell-cycle entry. Interestingly, inactivation of *ubc-25*, a gene encoding a highly conserved E2 ubiquitin-conjugating enzyme (UBC), resulted in quiescence defects during vulva and intestine development. Genetic and biochemical analyses indicated that *ubc-25* acts in a linear pathway with *cul-1* to control cell-cycle quiescence and that its activity negatively regulates steady-state CYE-1 abundance. Together, our studies suggest that these newly identified genes are important cell-cycle regulators during *C. elegans* development and the dysfunction of their human homologs may contribute to carcinogenesis.

## Materials and Methods

### *C. elegans* strains and culture

*C. elegans* were maintained at 20° as previously described (Brenner 1974), unless stated otherwise. Animals were examined using a Zeiss AxioImager microscope, AxioCam camera, and Axiovision software. Image cropping and annotations were performed using Adobe Photoshop and ImageJ software. The following strains were used in these studies: JK2868: *qIs56[lag-2*::*gfp]*V (Blelloch *et al.* 1999), KM166: *cye-1(eh10)/dpy-14(e188)*I (Brodigan *et al.* 2003), MH1829: *fzr-1(ku298) unc-4(e120)*II (Fay *et al.* 2002), MT6034: *lin-36(n766)*III (Thomas and Horvitz 1999), MT10430: *lin-35(n745)*I (Lu and Horvitz 1998), PD4667: *ayIs7[hlh-8*::*gfp]*IV (Corsi *et al.* 2000), RB1481: *ubc-25(ok1732)*I (this study), RG733: *wIs78[ajm-1*::*gfp + scm*::*gfp]*IV (Abrahante *et al.* 2003), SV326: *rtIs14[elt-2*::*GFP*; *osm-10*::*HT150Q]*IV (Fukushige *et al.* 1998; Saito *et al.* 2004), SV557: *cdc-14(he141)*II (Saito *et al.* 2004), VW22: *rrf-3(pk1426)*II; *lin-12(n950)*III; *lag-2(sa37)*V (this study), and VW198: *cyd-1(he112)/mIn1* II; *rtIs14[elt-2*::*GFP*; *osm-10*::*HT150Q]*IV (Boxem and van den Heuvel 2001).

### Analyses of VPC cell-cycle quiescence

The genome-wide RNA interference (RNAi) screen used the feeding method to generate loss-of-function phenotypes (Fraser *et al.* 2000; Timmons *et al.* 2001; Kamath *et al.* 2003). VW22: *rrf-3(pk1426)*; *lin-12(n950)*; *lag-2(sa37)* triple mutant animals were used for their RNAi hypersensitivity (Simmer *et al.* 2003) (Supporting Information, Figure S1) and improved viability (Tax *et al.* 1994; Clayton *et al.* 2008) compared with *lin-12(n950)* mutant animals. Primary screening of the Ahringer RNAi feeding library (Fraser *et al.* 2000; Kamath *et al.* 2003) initiated by seeding approximately 10 L1-synchronized (Hong *et al.* 1998; van den Heuvel and Kipreos 2012) VW22 animals on the RNAi bacteria. Following 8 days of growth at 15°, the F1 generation was screened for the presence of adult animals displaying the enhancer of *lin-12(gf)* multivulva (Elm) phenotype of greater than 6 pseudovulvae. The appearance of a single Elm animal was considered a positive result. Thus, the Elm frequency was not determined during the screen because the vast majority of positive hits consisted of a single Elm adult amid an undetermined number of Muv (non-Elm) adults. RNAi clones found to induce lethality or fertility defects were reanalyzed by exposing approximately 100 synchronized L1 animals to the feeding RNAi clone at 15° and examining the adult worms after 5 d for the Elm phenotype. A total of 1004 RNAi clones were initially found to produce the Elm phenotype and retested. As previously described (Saito *et al.* 2004), a defect of cell-cycle quiescence allows ectopic cell divisions that produce extra VPCs. Thus, we examined the number of VPCs at the L2-to-L3 molt using Nomarski optics to distinguish between defects in cell fate determination and cell-cycle quiescence. The identities of the RNAi-targeted genes were confirmed by sequencing. 108 RNAi clones (two separate clones targeting *mdt-1.1*/*sop-3* were isolated) were subsequently determined to disrupt cell-cycle quiescence.

### Quantification of intestinal nuclei

The *elm* gene regulation of cell cycles during intestine development was examined using standard RNAi feeding procedures and genetic mutations when appropriate. For all experiments examining intestinal nuclei number, visualization of intestinal nuclei was aided by *rtIs14[elt-2*::*GFP*; *osm-10*::*HT150Q]*IV, which expresses an irrelevant neuron-specific transgene, *osm-10*::*HT150Q*, in addition to an integrated *elt-2*::GFP reporter. For experiments using RNAi, L4 animals were transferred to RNAi-inducing bacteria and intestinal nuclei of L4-to-young adult aged F1 self-progeny (n ≥ 10) were scored after 4−5 d at 20°. The RNAi clone targeting *unc-73* was used as the negative control for all experiments. For assays to measure genetic enhancement of intestinal nuclei production, significance (*P*-value < 0.05) was determined by an unpaired two-tailed Student’s *t*-test comparing the double loss of activity to either single alone.

### ubc-25 mutation and transgene

The strain harboring the *ubc-25(ok1732)* mutation, RB1481, was obtained from the Caenorhabditis Genetics Center and outcrossed four generations. The *ok1732* ∼1.2kb deletion was confirmed by polymerase chain reaction using the primers *ubc-25*-5′ATG+Nhe, 5′-GCTAGCATGGCGTGTCTTCGAAAACTAAAAGAAGAC-3′; *ubc-25*-3′-1, 5′-CCTGATAAAACGCGAGTTTCAAAACAGCTCAC-3′; and *ubc-25*-3′-2, 5′-CATCGTCAACTTCTCCATCTCCAGC-3′. The mCherry::UBC-25 transgene contains ~1.6-kb promoter sequence upstream of a translational fusion between mCherry (pAA64; Audhya *et al.* 2007) and UBC-25 coding sequences. The *ubc-25* promoter and coding sequences were amplified by polymerase chain reaction from genomic DNA using the primer sets P*ubc-25*-5′+Bam (5′-GGATCCTGTAACCCTCATTTTTGCTCTATGTATC-3′) to P*ubc-25*-3′+Age (5′-GGTACCTCTTCTGATTTTCGCTACC-3′) and *ubc-25*-5′ATG+Nhe to UBC-25-3′+Nhe (5′-GCTAGCTTATCCTTCTGTTTTTGGAGGT-3′), respectively. The UBC-25 coding sequence was inserted in-frame in place of the mCherry termination codon using an *Nhe*I site inserted immediately upstream of the *let-858* 3′ untranslated region. The promoter was subsequently cloned upstream of mCherry using *Bam*HI and AgeI to generate the P*ubc-25*::mCherry::UBC-25 expression plasmid.

### 4D cell lineage analyses

Wild-type and *ubc-25(ok1732)* embryos were imaged at 25° using four-dimensional microscopy essentially as previously described (Schnabel *et al.* 1997). Both strains contained *rtIs14* for visualization of the intestinal nuclei. Images of embryos were transformed into four-dimensional cell lineages and analyzed using SimiBiocell software (Simi Reality Motion Systems GmbH).

### Western blot analyses

Sodium dodecyl sulfate polyacrylamide gel electrophoresis followed by western blotting was used to measure steady-state expression of CYE-1. For each sample, 50-100 gravid adult animals were boiled in 2X loading buffer and proteins separated on 4–15% precast gradient sodium dodecyl sulfate polyacrylamide gel electrophoresisgels (BioRad). Samples were transferred to nitrocellulose membrane and probed using anti-CYE-1 antibodies (1:2000 dilution; Brodigan *et al.* 2003). Anti-α-tubulin monoclonal antibody (DM1A; Sigma-Aldrich) was used at 1:5000 dilution. Supersignal (Thermo Scientific) was used for developing anti-α-tubulin and anti-CYE-1 western blots. The relative CYE-1 protein levels were quantified from scanned films using ImageJ.

## Results

### A genome-wide RNAi screen identified 107 cell-cycle quiescence regulators

**Figure 1:**
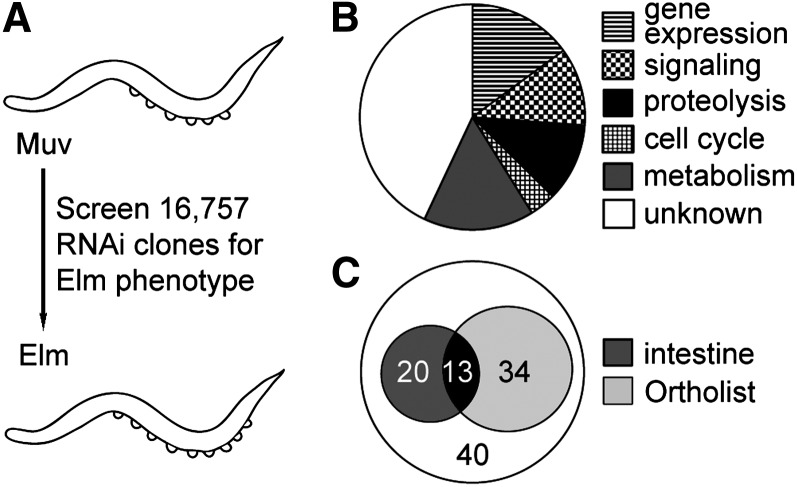
Conserved negative cell-cycle regulators were identified in the Elm screen. (A) Schematic diagram of genome-wide RNA interference (RNAi) screen. RNAi feeding clones were independently tested for the ability to transform the progeny of *lin-12(gf)* multivulva (Muv) animals into Elm animals that display greater than six pseudovulvae. (B) Pie chart illustrating the distribution of the predicted functions for the 107 genes identified by the Elm screen. (C) Venn diagram of 107 genes identified by the genome-wide RNAi screen as potential cell-cycle quiescence regulators. Thirteen genes overlap between the 33 genes also necessary for restricting cell cycles in the intestine and the 47 genes listed on OrthoList.

We previously conducted a forward genetic screen for the Elm phenotype and identified several previously unrecognized components of a developmental network controlling cell-cycle quiescence in *C. elegans* (Saito *et al.* 2004; Clayton *et al.* 2008). The Elm screen relies on the *lin-12*/Notch gain-of-function mutation to direct differentiation of VPCs into obvious ventral protrusions called pseudovulvae (Greenwald *et al.* 1983). Since wild-type animals produce only six VPCs (Sulston and Horvitz 1977) and each VPC can give rise to a single pseudovulva, *lin-12(n950)* animals display a maximum of six pseudovulvae (Greenwald *et al.* 1983). In contrast, Elm mutant animals produce ectopic VPCs through either extra cell divisions (Saito *et al.* 2004) or transformations of cell identities (Alper and Kenyon 2001) and display greater than six pseudovulvae. Here we conducted an RNAi-based, genome-wide examination of the genetic network controlling VPC cell-cycle quiescence using *rrf-3(pk1426)*; *lin-12(n950)*; *lag-2(sa37)* triple mutant animals (Figure S1) to screen for the Elm phenotype. Each of the 16,757 RNAi clones contained within the feeding library (Fraser *et al.* 2000; Kamath *et al.* 2003) was individually tested for the ability to induce the production of greater than six pseudovulvae (Figure 1A). The RNAi experiments found to produce the Elm extra pseudovulvae phenotype were further scrutinized for ectopic VPC divisions during larval development, which would indicate a defect of cell-cycle quiescence (Hong *et al.* 1998). The inhibition of 107 genes by RNAi (Table S1), less than 1% of the total genes predicted within the genome, produced the Elm phenotype as a result of a cell-cycle quiescence defect.

The 107 genes represent putative components of a regulatory network controlling cell-cycle quiescence during development. Notably, the identified genes included the previously characterized cell-cycle quiescence regulators *cdc-14*/Cdc14 (Saito *et al.* 2004), *cki-1*/p27 (Hong *et al.* 1998; Feng *et al.* 1999; Fukuyama *et al.* 2003), *cki-2*/p27 (Buck *et al.* 2009), *cul-1*/Cul1 (Kipreos *et al.* 1996), and mediator subunits *mdt-1.1*/*sop-3*/MED1 and *mdt-12/dpy-22*/MED12 (Clayton *et al.* 2008). The identification of multiple genes known to play roles in cell-cycle quiescence validated this screening approach. To begin a functional evaluation of the regulatory network, we considered the sequence conservation and tissue specificities of the 107 genes. The genes were categorized based on the conservation of their amino acid sequences into six general classes: (1) regulated proteolysis, (2) gene expression, (3) metabolism, (4) signal transduction, (5) cell cycle or (6) unknown, a group consisting of members exhibiting conservation with either uncharacterized genes or no recognizable conservation (Figure 1B and Table S1). We determined that 70 of the 107 genes (65%) were represented by recognizable human orthologs (Table S1). In fact, 47 of these genes appear on the *C. elegans*-human ortholog compendium, OrthoList (Shaye and Greenwald 2011). The conservation of the genes selected by the Elm phenotype screen may indicate an overall conservation of the mechanisms controlling cell-cycle quiescence between *C. elegans* and humans.

We next investigated whether the 107 genes identified as cell-cycle regulators in VPCs were required for cell-cycle quiescence during the development of an unrelated tissue, the intestine. The entire intestine develops from a single cell that undergoes multiple rounds of cell divisions throughout embryonic and larval development; however, larval development incorporates unusual cell cycles resulting in karyokinesis without cytokinesis and polyploidy (Mcghee 2007). The exceptional development of the intestine further enhances the detection of cell-cycle defects (Boxem and van den Heuvel 2001). We found that the RNAi-mediated inhibition of 33 genes disrupted cell-cycle regulation as shown by the production of extra intestinal nuclei (Figure 1C and Table S1), indicating that these 33 genes act broadly in multiple tissues to control cell cycles.

### ubc-25 activity promotes cell-cycle quiescence

*ubc-25* is one of 13 genes identified by the screen that both appears on the Ortholist and acts in intestine and vulva development (Figure 1C and Table S1). Ubiquitin-conjugating enzymes such as UBC-25 transfer ubiquitin to a target protein substrate, usually in conjunction with an E3 ubiquitin ligase, to regulate protein activity, localization, interaction and stability (Kipreos 2005). Accordingly, *ubc-25* was examined as an example of a potentially fundamental regulator of cell-cycle quiescence during metazoan development. UBC-25 exhibited high amino acid sequence conservation with UBE2Q2, a metazoan specific UBC implicated in cancer (Schulze *et al.* 2003; Melner *et al.* 2006; Maeda *et al.* 2009). Although we identified *C. elegans ubc-25* as a regulator of VPC cell-cycle quiescence, an analyses of VPC number at the L2-to-L3 molt indicated that extra cell divisions were rare in the *ubc-25* loss-of-function animals (Table 1). The weak cell-cycle quiescence defect was significantly enhanced by concurrent loss of *lin-35* Rb activity. *ubc-25(RNAi)* animals also displayed a variable intestine defect that frequently lead to the observation of extra nuclei at the completion of larval development (38.3 ± 6.7, n = 20) compared with control RNAi animals (32.2 ± 1.3, n = 15) (Table S1). Because of the greater penetrance of the intestinal phenotype, we focused our analyses of the role of *ubc-25* in controlling cell cycles during intestine development.

**Table 1:** Enhanced cell-cycle quiescence defect of *ubc-25(lf)*; *lin-35(lf)* animals

Genotype	RNAi*^a^*	% Elm Animals*^b^*	n
Wild type	*unc-73*	0	65
Wild type	*lin-35*	0	90
Wild type	*ubc-25*	2	46
*lin-35(n745)*	*unc-73*	0	27
*lin-35(n745)*	*ubc-25*	23	26
*ubc-25(ok1732)*	*unc-73*	6	109
*ubc-25(ok1732)*	*lin-35*	22	96

RNAi, RNA interference; VPC, vulval precursor cell.

a *unc-73(RNAi)* is used as the negative control.

b The Elm cell-cycle quiescence defects were scored using the more sensitive measure of extra VPC production by directly examining animals at the L2-to-L3 molt.

**Figure 2:**
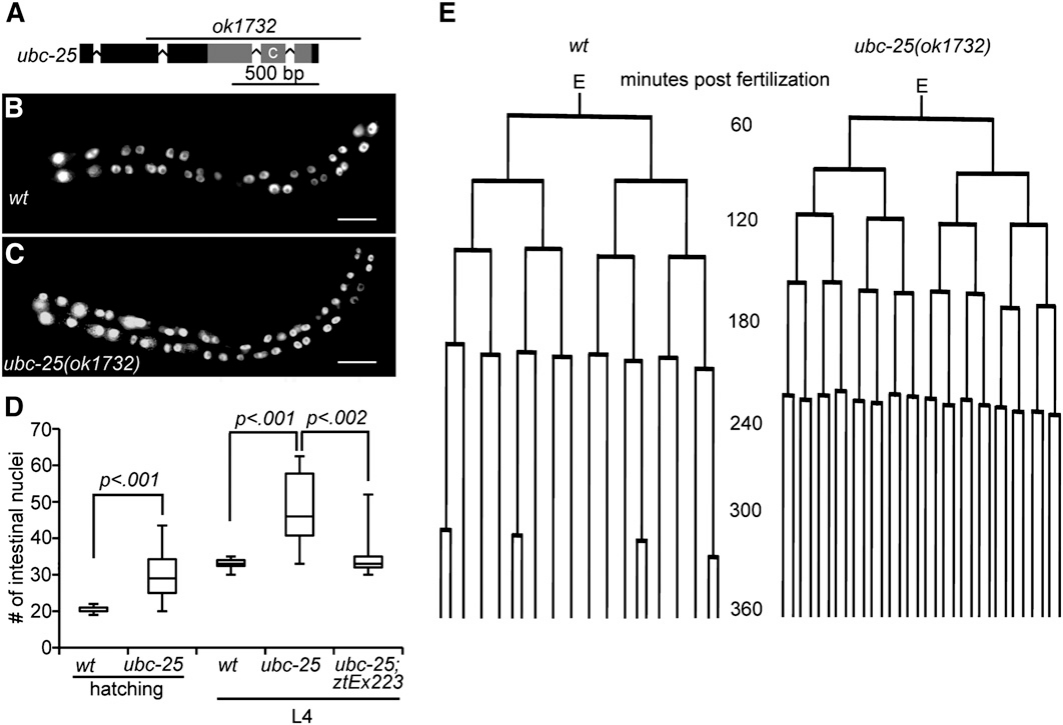
*ubc-25* is a negative regulator of intestinal cell cycles. (A) Schematic diagram of the *ubc-25* locus. Exons and introns are indicated by boxes and connecting lines, respectively. Gray shading indicates sequences encoding the E2 domain. Location of catalytic cysteine is labeled “C.” Region deleted by *ok1732* mutation is delineated by labeled line. (B) Image of wild-type L2-aged animal with intestinal nuclei highlighted by the *elt-2*::*gfp* transgene within *rtIs14*. (C) Image of typical *ubc-25(ok1732)* age-matched animal displaying extra intestinal nuclei. Scale bars indicate 20 μm. (D) Quantification of intestinal nuclei of the indicated genotypes and ages. *ztEx223* is an extrachromosomal array containing the P*ubc-25*::mCherry::UBC-25 expression plasmid. The median, 25%, and 75% quartiles are shown as centerline and lower and upper box edges, respectively. The whiskers indicate the total range of values (n ≥ 15). (E) Comparison of representative cell lineages observed for the intestinal E lineages of (left) wild type (wt; n = 2 embryos) and (right) *ubc-25(ok1732)* (n = 5 embryos) animals. The wild-type lineage is indistinguishable from lineages previously observed in wild type (Yan *et al.* 2013).

We obtained a strain harboring a predicted null mutation, *ubc-25(ok1732)*, that deletes the conserved ubiquitin-conjugating domain (Figure 2A). Although the *ubc-25(ok1732)* animals appear superficially normal, growth at 25° resulted in a significant reduction of self-brood size (64.9 ± 15.5 *vs.* 190.6± 32.5 for wild type) and increase of embryonic lethality (69.8% *vs.* 1.6% for wild type) (Table S2). This decrease of fertility and fecundity is consistent with *ubc-25* functioning in an essential process, such as cell-cycle regulation. However, the activity of *ubc-25* is not ubiquitously required for cell-cycle quiescence because no cell-cycle defects were observed in the M, V, and somatic Z lineages of *ubc-25(ok1732)* larvae (Table S3). Importantly, the *ubc-25(ok1732)* mutant animals displayed extra VPCs (Table 1) and intestinal nuclei (Figure 2, B and C), confirming the role of *ubc-25* in limiting cell cycles during development of these diverse tissues.

Quantification of intestinal nuclei at the beginning and end of larval development demonstrated that the extra nuclei of *ubc-25(ok1732)* arise earlier during embryogenesis (Figure 2D). Therefore, we determined the embryonic division patterns of the E cell and its descendents that give rise to the intestine. This cell lineage analysis revealed that the time between consecutive mitoses was significantly decreased within *ubc-25(ok1732)* embryos (Table S4). Thus, five rounds of cell divisions are completed within roughly the same period that wild-type E lineages complete four rounds (Figure 2E). Therefore, *ubc-25(ok1732)* animals displayed a significant increase of intestinal nuclei by the completion of embryogenesis. In contrast, during larval development the intestinal nuclei normally undergo a series of specialized cell divisions (McGhee 2007), and the proportion of dividing intestinal nuclei were indistinguishable between wild-type and *ubc-25(ok1732)* mutant larvae (60% and 64%, respectively). Thus, the embryonic and larval extra cell-cycle defects during intestine and vulva development, respectively, indicated that the primary developmental role of *ubc-25* is to inhibit cell-cycle entry and/or promote cell-cycle quiescence.

### ubc-25 is widely expressed during development

**Figure 3:**
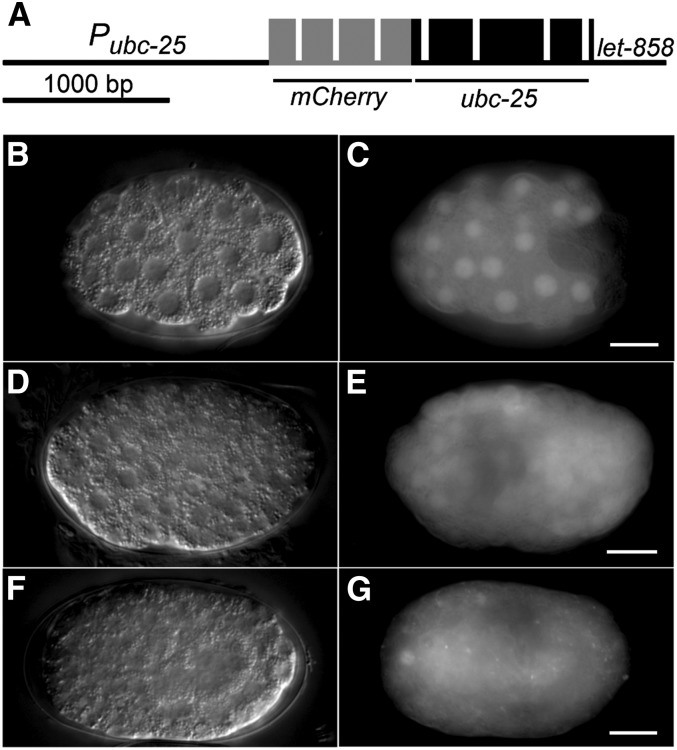
Expression of a *mCherry*::*ubc-25* reporter. (A) Schematic diagram of the *mCherry*::*ubc-25* transgene. Exons encoding mCherry and UBC-25 are indicated as gray and black boxes, respectively. (B, D, F) Nomarski and (C, E, G) epifluorescence images of *ztEx223* containing transgenic animals display the expression of the mCherry::UBC-25 fusion protein. Embryos of approximately (B, C) 50 cells, (D, E) 100 cells, and (F, G) bean stage are shown. Scale bars indicate 10 μm.

To provide further confirmation of a role for *ubc-25* in regulating cell cycles and to determine its spatiotemporal expression pattern, we produced a transgene expressing a translational fusion between mCherry and UBC-25 (Figure 3A). Although the effect on brood size or embryonic lethality was not examined, expression of this *ubc-25(+)* transgene in *ubc-25(ok1732)* mutant animals restored the normal number of intestinal nuclei (Figure 2D), further confirming that loss of *ubc-25* activity is responsible for the cell-cycle defects. The expression of *ubc-25* as indicated by the mCherry::UBC-25 chimeric protein was widespread during embryogenesis (Figure 3, B−G), consistent with an earlier report (Schulze *et al.* 2003). Interestingly, mCherry::UBC-25 appeared to localize within nuclei during early embryogenesis when cells are rapidly dividing (Figure 3C) but progressively becomes distributed throughout the cell later in embryogenesis when the frequency of cell cycles are reduced (Figure 3G). The ubiquitous expression of mCherry::UBC-25 suggests that although *ubc-25* activity is rate limiting in select tissues, other processes within the network controlling cell-cycle quiescence may conceal loss of *ubc-25* activity in some cell types.

### The integration of ubc-25 activity within the regulatory network

**Figure 4:**
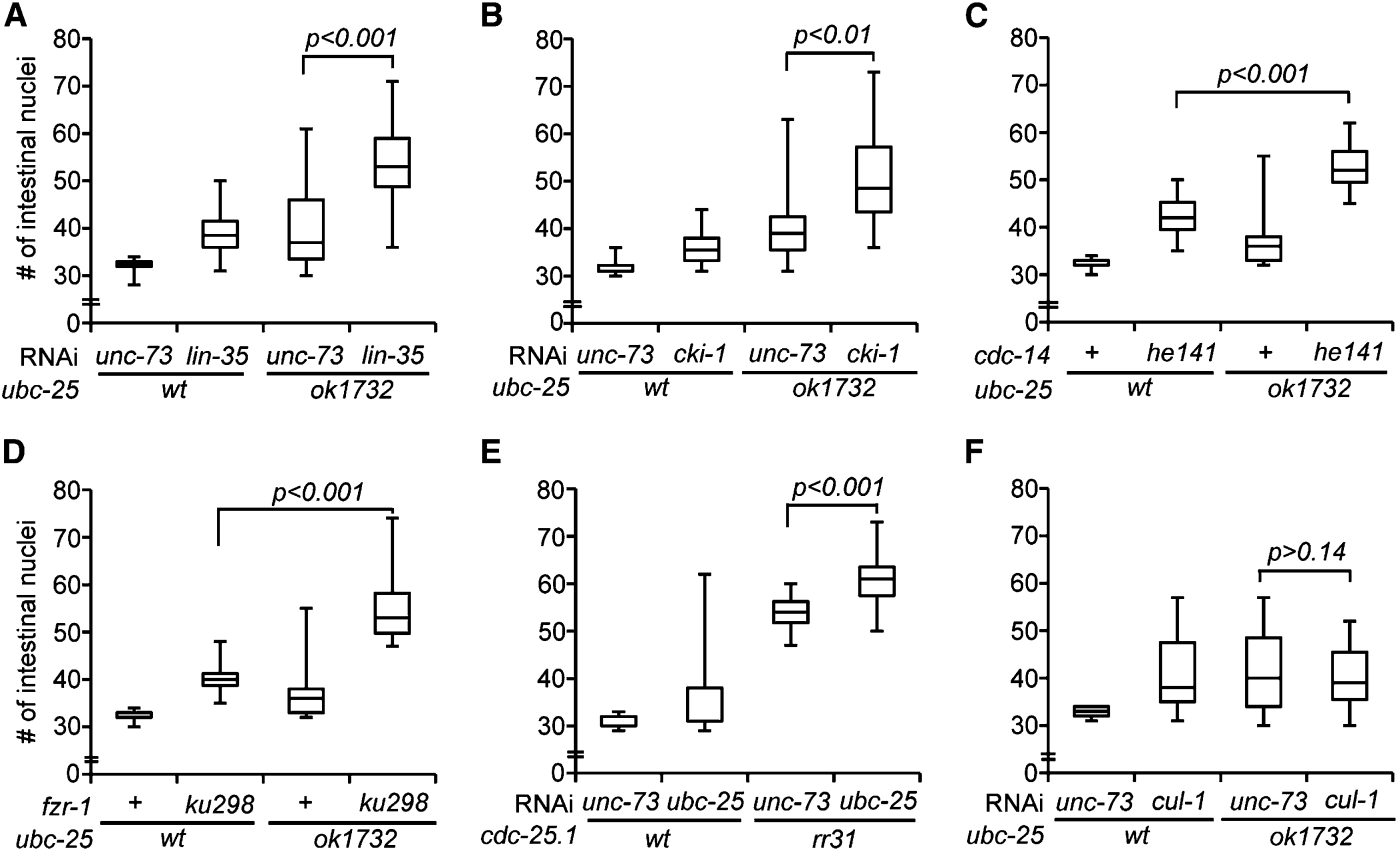
Genetic analyses indicate that *ubc-25* acts with *cul-1*. Box and whisker plots presenting quantification of intestinal nuclei in *rtIs14* animals deficient for *ubc-25* activity in combination with (A) *lin-35(RNAi)*, (B) *cki-1(RNAi)*, (C) *cdc-14(he141)*, (D) *fzr-1(ku298)*, (E) *cdc-25.1(rr31)*, and (F) *cul-1(RNAi)*. *unc-73(RNAi)* is used as a negative control. RNA interference treatment or second genetic mutation is indicated above the horizontal line that indicates the common genetic background indicated below. Statistical significance is indicated for animals carrying the double mutation combination compared to the greater of the two single mutations alone. n ≥ 19 animals examined.

Several genetically distinct pathways have been described that act in parallel to control G_1_/S progression in the intestine (Kipreos *et al.* 1996; Hong *et al.* 1998; Boxem and van den Heuvel 2001; Fay *et al.* 2002; Kostic and Roy 2002; Saito *et al.* 2004; Grishok and Sharp 2005; Buck *et al.* 2009; Roy *et al.* 2011). To place *ubc-25* activity within a specific genetic pathway, we determined whether loss of *ubc-25* activity enhanced the cell-cycle defects caused by disruptions to these known pathways. The combination of *ubc-25(ok1732)* with *lin-35*/Rb, *cki-1*/p27, *cdc-14*/Cdc14 or *fzr-1*/Cdh1 loss of activity (Figure 4, A–D, respectively), or *ubc-25(RNAi)* with a *cdc-25.1*/Cdc25 gain-of-function mutation (Figure 4E) resulted in a significant increase of intestinal nuclei number. The enhancement of the loss-of-function phenotypes suggested that the processes mediated by these genes function in parallel to *ubc-25*. In contrast, phenotypic enhancement was not observed between *ubc-25(ok1732)* and *cul-1(RNAi)* (Figure 4F), suggesting that these genes act within the same pathway or complex. *cul-1*/Cul1 encodes a component of an SCF (Skp1-Cul1-Fbox) ubiquitin ligase (E3 enzyme) complex whose mammalian homologs control the abundance of cyclin E to inhibit cell-cycle progression (Dealy *et al.* 1999; Wang *et al.* 1999). Together, these genetic interactions are consistent with *ubc-25* acting in conjunction with the SCF complex to regulate G_1_/S progression.

**Figure 5:**
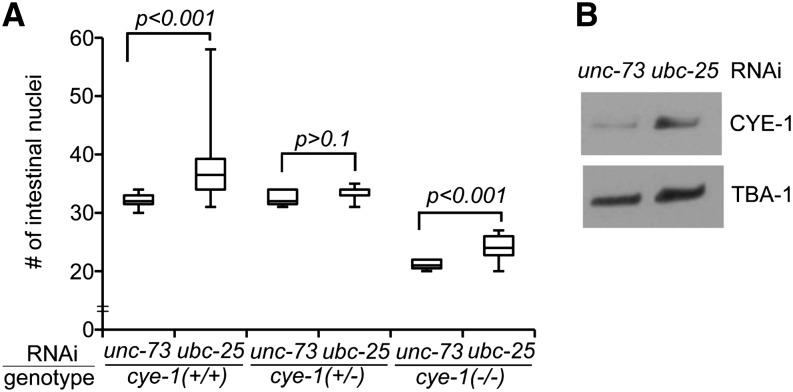
*ubc-25* is a negative regulator of *cye-1*. (A) Box and whisker plot presenting the effect of *ubc-25(RNAi)* on intestinal nuclei number in self-progeny of *cye-1(+/−)*; *rtIs14* hermaphrodites. For each *cye-1* experimental pair, connected by horizontal lines, the RNAi treatment of either *ubc-25* or the negative control *unc-73* is indicated above. n ≥ 15 for each condition. (B) Western blot illustrating increased expression of CYE-1 upon inhibition of *ubc-25* activity by RNAi. Steady-state expression of CYE-1 is increased at least threefold (n = 3). TBA-1/α-tubulin is used as a loading and normalization control.

Because the *cul-1*-mediated pathway likely regulates the cell cycle by targeting activities that promote cell-cycle progression, we examined *cye-1*/cyclin E as a potential downstream target of *ubc-25*. To determine the dependence of the *ubc-25(lf)* extra intestinal nuclei phenotype on *cye-1* activity, we varied the *cye-1(+)* dosage using the *cye-1* null allele, *eh10* (Brodigan *et al.* 2003). Heterozygous animals, *cye-1(eh10/+)*, were treated with *ubc-25(RNAi)*, and the numbers of intestinal nuclei were compared between the self-progeny. In wild-type *cye-1(+/+)* progeny, *ubc-25(RNAi)* produced extra intestinal nuclei similar to the *ubc-25(ok1732)* allele. In contrast, the *cye-1(eh10/+)* progeny displayed a wild-type average (Figure 5A), indicating that the extraintestinal nuclei phenotype is dependent on *cye-1(+)* dosage. Interestingly, heterozygous *cye-1(eh10/+)* hermaphrodites give rise to viable but sterile *cye-1(eh10)* homozygous offspring that develop into larvae due to the persistence of maternally contributed *cye-1* activity (Fay and Han 2000; Brodigan *et al.* 2003). These *cye-1(eh10)* homozygous progeny allow us to test the prediction that the function of *ubc-25* is to down regulate *cye-1* activity. In fact, the loss of *ubc-25* function within the homozygous *cye-1(eh10)* progeny produced a weaker cell-cycle defect presumably due to the increased stability of the maternally contributed *cye-1* activity (Figure 5A). A similar partial rescue of cell-cycle defects was described during vulva development of *cye-1*; *cul-1* double-mutant animals (Fay and Han 2000). Together, the genetic data support a model wherein *ubc-25* controls intestinal cell divisions through the inhibition of *cye-1* activity.

To test the hypothesis that *ubc-25* inhibits cell cycles through the control of CYE-1 protein expression, we determined the steady-state level of CYE-1 in *ubc-25*-deficient animals. *ubc-25(RNAi)*-treated animals displayed increased CYE-1 compared with the negative control animals (Figure 5B), confirming that *ubc-25* activity negatively regulates CYE-1 expression. Together, these biochemical and genetic results demonstrate that *ubc-25* activity inhibits *cye-1* function and that the cell-cycle quiescence defects of *ubc-25* deficient animals are likely due to enhanced CYE-1 activity.

To identify genes acting with *ubc-25* to regulate cell cycles, we applied a complementary biochemical approach. A yeast two-hybrid screen using UBC-25 as bait was used to identify potential UBC-25 co-factors, regulators, or targets (Figure S2). Remarkably, the screen of over 10^7^ interactions isolated 30 clones that identified a single gene, C30H7.2, encoding an ortholog of a human 44-kD endoplasmic reticulum chaperone protein. Based on RNAi analyses, C30H7.2 was found to be dispensable for cell-cycle quiescence (Figure S2). Although a physical interaction between UBC-25 and C30H7.2 may play a significant role in an alternative physiological process, the characterization of this process is outside the focus of our cell-cycle regulation studies.

### Genetic redundancies within the regulatory network

We noted that loss of *ubc-25* activity did not result in a strong cell-cycle phenotype, particularly when compared with the SCF components, *cul-1* and *lin-23*, whose loss of functions result in stronger and more widespread hyperplasia (Kipreos *et al.* 1996, 2000). Thus, we searched for evidence of parallel or overlapping functions of genes within the regulatory network.

**Figure 6:**
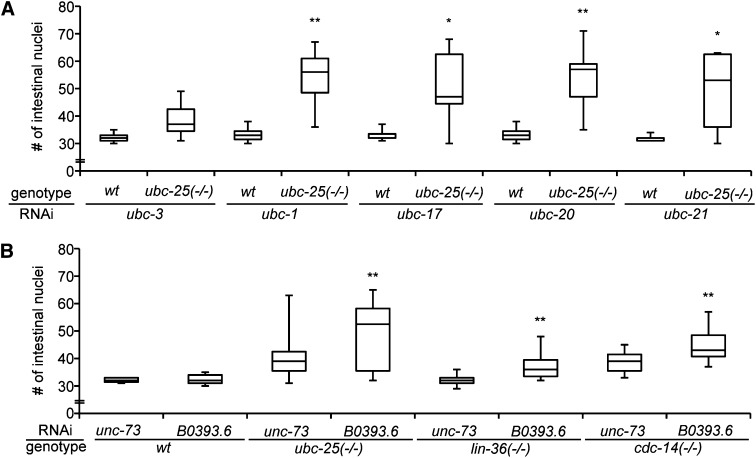
Genetic redundancy ensures strict control of cell-cycle quiescence. (A) Box and whisker plot presenting the effect of RNA interference (RNAi)-mediated inhibition of *ubc-1*, *ubc-17*, *ubc-20*, and *ubc-21* on intestinal nuclei number in *wt* and *ubc-25(ok1732)* animals. *ubc-3(RNAi)* illustrates an example of an *ubc* gene that does not display redundancy. (B) *B0393.6(RNAi)* significantly enhanced the number of intestinal nuclei of *ubc-25(ok1732)*, *lin-36(n766)* and *cdc-14(he141)* mutant animals, indicating a fourth distinct genetic pathway promoting cell-cycle quiescence. Statistical significance was determined by comparing test RNAi animals to the RNAi of the negative control gene, *unc-73*, using the two-tailed Student *t*-test (*P < 0.05 and **P < 0.01). n ≥ 15 animals examined for each condition.

We first investigated potential compensatory activities between the 22 members of the *C. elegans ubc* family. The phenotypes resulting from *ubc* gene RNAi were compared between wild-type and *ubc-25(ok1732)* mutant animals to determine whether the loss of two *ubc* activities produced an enhanced cell-cycle quiescence phenotype (Table S5). The majority of *ubc*-targeting RNAi clones did not enhance the extra intestinal nuclei defect. However, inhibition of *ubc-1*, *ubc-17*, *ubc-20*, or *ubc-21* by RNAi resulted in significant increases of intestinal nuclei in *ubc-25(ok1732)* animals but no discernible effect was observed in wild-type animals (Figure 6A). Although the relationships between these other *ubc* genes have not been explored further, we can conclude that *ubc-1*, *ubc-17*, *ubc-20*, and *ubc-21* can contribute cell-cycle regulatory activity in the absence of *ubc-25* function.

We next examined the other components of the developmental network for potentially redundant activities. All 107 Elm screen-positive RNAi clones were analyzed for enhancement or suppression of intestinal phenotypes in strains harboring the *ubc-25(ok1732)*, *cdc-14(he141)*, *lin-36(n766)*, or *cyd-1(he112)* mutation. Loss of *lin-35* function results in transgene silencing (Hsieh *et al.* 1999). Therefore, we used *lin-36(n766)* animals in the analyses because *lin-36* acts with *lin-35* to regulate cell cycles but *lin-36* is not necessary for maintenance of transgene expression (Boxem and van den Heuvel 2002). In total, 25 of the 107 RNAi clones significantly enhanced the extra intestinal nuclei of at least one test genotype (Table S6). Specifically, the extraintestinal nuclei phenotypes resulting from *ubc-25(ok1732)*, *lin-36(n766)*, and *cdc-14(he141)* mutations were enhanced by 15, 8, and 11 RNAi clones, respectively. In addition, RNAi-mediated inhibition of three genes (B0393.6, *cdc-14*, or *ubc-25*) partially suppressed the proliferation defects caused by the *cyd-1(he112)* mutation. Importantly, *ubc-25(RNAi)* enhanced the *lin-36(n766)* and *cdc-14(he141)* mutations and both *cdc-14(RNAi)* and *cki-1(RNAi)* enhanced the *ubc-25(ok1732)* and *lin-36(n766)* mutations (Table S6), corroborating our earlier results.

We used the grouping by genetic enhancement to predict regulatory organization within the network. In addition to the aforementioned expected results, inhibition of two genes, *gmn-1* and *hda-2*, enhanced the phenotypes of *cdc-14(he141)* and *ubc-25(ok1732)* but not *lin-36(n766)*, suggesting that these genes may act within the *lin-35*-mediated process. Similarly, inhibition of nine genes (*cul-1*, *dcp-66*, F19B10.6, F49E11.7, K09F6.9, *ppk-1*, Y54E10BR.3, Y71H2AM.4, and ZK1236.9) enhanced the mutant phenotype of either *lin-36(n766)* or *cdc-14(he141)* without effecting the *ubc-25(ok1732)* defect. This approach also provides evidence for a previously unrecognized pathway that negatively controls cell-cycle entry. We found that B0393.6, a gene encoding a RING domain protein (Kipreos 2005), was uniquely able to enhance intestinal nuclei in all test strains (Figure 6B). Integrating the genetic enhancement results for these 107 genes provides a framework for future studies focusing on pathway interactions in the maintenance of cell-cycle quiescence.

## Discussion

We used a genome-wide RNAi screen to uncover the genes that constitute a developmental network controlling cell divisions in *C. elegans* and uncovered 100 genes not previously known to contribute to cell-cycle quiescence. The Elm phenotype screen proved to be a sensitive and reliable indicator of cell-cycle defects leading to the production of extra VPCs during development. For example, the screen successfully identified *ubc-25* despite the fact that even the null mutation of *ubc-25* rarely caused extra VPC divisions. Despite the screen sensitivity, some genes acting within the network likely remain undiscovered because approximately 14% of loci are not represented within the library and some genes are refractory to RNAi inhibition (Fraser *et al.* 2000; Gonczy *et al.* 2000; Kamath *et al.* 2003). Regardless, the identification of these network components constitutes considerable progress toward a comprehensive understanding of the regulatory interactions that define the network controlling cell cycles.

### Elaborating a complex cell-cycle regulatory network

Of the 107 identified components of the developmental network, only 33 genes appear to be nematode specific. Thus, the majority of genes revealed by the screen may perform conserved functions throughout metazoans as components of the machinery coordinating cell cycles with development. The conserved genes implicate specific processes as crucial for cell-cycle control. For example, four genes that control gene expression specifically through regulation of chromatin were identified by the screen as necessary for cell-cycle quiescence: *hda-2* (Shi and Mello 1998), *jhdm-1* (Maures *et al.* 2011), *dcp-66* (Poulin *et al.* 2005), and *egl-27* (Herman *et al.* 1999; Solari *et al.* 1999) encode a histone deacetylase, a histone demethylase and the p66 and MTA1 components of the nucleosome remodeling and deacetylation (*i.e.*, NuRD) complex, respectively. Together with the previously described transcriptional regulators, *lin-35/*pRb (Lu and Horvitz 1998), *mdt-1.1*/MED1 and *mdt-12*/MED12 (Clayton *et al.* 2008), these genes highlight the important activities that can be revealed for general regulators of transcription by examining tissue- and developmental stage-specific phenotypes. Intriguingly, our genetic interaction data indicate functional cooperation between *lin-35*/pRb, *hda-2*/HDAC1 and the *C. elegans* homolog of the dual-function protein, *gmn-1*/Geminin (Luo and Kessel 2004; Yanagi *et al.* 2005). In human cell lines, pRB and HDAC1 control cell cycles through cyclin E expression (Brehm *et al.* 1998; Magnaghi-Jaulin *et al.* 1998). Similarly, Geminin acts during development to promote the transition from a proliferative state to differentiation (Del Bene *et al.* 2004; Luo and Kessel 2004), possibly through a mechanism involving chromatin acetylation (Yellajoshyula *et al.* 2011). As these examples illustrate, the careful analyses of the newly identified genes may result in crucial observations leading to a better understanding of the complex regulatory network coordinating cell cycles with development.

### The network employs parallel circuits that converge on regulation of CDK2 activity

**Figure 7:**
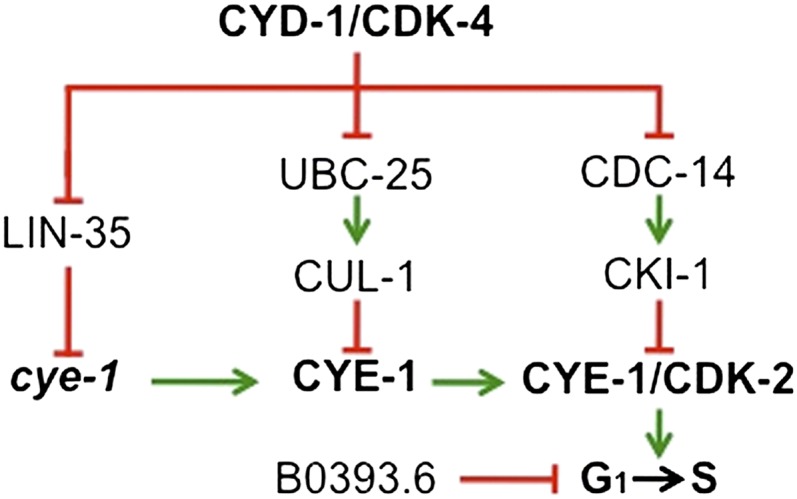
Model illustrating cooperation within the cell-cycle quiescence regulatory network. Genetic analyses suggest that at least four genetically distinct pathways collaborate to control cell cycles during development. As described in the text, three pathways mediated by LIN-35/pRb, UBC-25/UBE2Q2, and CKI-1/p27 regulate *cye-1*/cyclin E activity at the level of transcription, protein stability and activity, respectively. The mechanism through which B0393.6/RNF182 controls cell cycles remains undefined.

Three independent pathways controlling cell-cycle quiescence have been connected to the regulation of cyclin E/CDK2 activity in *C. elegans* (Figure 7). First, *lin-35*/pRb inhibits transcription of *cye-1* (Grishok and Sharp 2005; Kirienko and Fay 2007; Grishok *et al.* 2008). Second, p27 family members inhibit the CYE-1/CDK-2 complex (Hong *et al.* 1998; Boxem and van den Heuvel 2001; Fukuyama *et al.* 2003; Buck *et al.* 2009). Third, our data demonstrate that *ubc-25* inhibits *cye-1* activity, likely through CUL-1-mediated ubiquitinylation and subsequent proteolysis of CYE-1 protein. Lastly, a potential fourth process involving B0393.6 inhibits cell cycles through a currently unexplored mechanism. These processes are interesting in light of the recent findings that the decision between cell-cycle entry and quiescence is determined by the activity of the cyclin E-partner, CDK2 (Spencer *et al.* 2013). In the human cell lines used in the study, the level of CDK2 activity at the end of the preceding mitosis must meet a threshold in order for the daughter cell to enter a new cell cycle. These results provide a molecular mechanism that is consistent with the models previously suggested for control of cell-cycle quiescence during the development of the Drosophila eye and *C. elegans* vulva by CDK inhibitors (de Nooij *et al.* 1996; Clayton *et al.* 2008).

The analyses presented here indicate that *ubc-25* acts as a negative regulator of steady-state CYE-1 expression, but it is not known whether this regulation is achieved through direct ubiquitinylation of CYE-1 by UBC-25. It is likely that UBC-25 ubiquitinylates a range of targets to regulate a variety of processes. In fact, *ubc-25* was previously recognized for roles not directly related to the cell cycle, such as promoting a Ras-mediated cell-fate decision (Rocheleau *et al.* 2008) and maintaining neuromuscular homeostasis (Schulze *et al.* 2003). It would be interesting to determine whether the putative interaction partner, C30H7.2, acts with UBC-25 in these alternative processes

Our genetic analyses provide insights into the regulatory mechanisms that may explain the relatively mild loss of function phenotypes observed when individual components are inactivated. First, other genes within a family may provide redundant activity. For example, 22 *ubc* genes are encoded in the *C. elegans* genome and we demonstrated that 4 genes, *ubc-1*, *ubc-17*, *ubc-20*, and *ubc-21*, could restrict intestinal cell cycles in the absence of *ubc-25* activity. However, because the UBC-25/UBE2Q2-related proteins possess an amino-terminal extension that may confer unique regulatory or substrate specificities (Jones *et al.* 2002; Melner *et al.* 2006), it is not known whether these four genes acted interchangeably with *ubc-25* or through a distinct compensatory mechanism. Second, the strict regulation of cell cycles is the collaborative result of independent pathways. In the specific case of intestinal cell cycles, the loss of *ubc-25* activity disturbs one regulatory mechanism that inhibits *cye-1* activity, but the parallel pathways remain intact and are collectively able to promote cell-cycle quiescence in the majority of cases. As a consequence of the multiple pathways acting in concert, the cell-cycle defects increase in severity upon disruption of two or more parallel pathways. Therefore, *ubc-25* illustrates the key concept that studies of regulatory networks need to consider: when multiple pathways cooperate to achieve robust control over a process, the loss of a single pathway may yield a weaker than expected phenotype.

The function of human UBE2Q2, alternatively designated as LOC92912 or UBCi, is not currently established. UBE2Q2 was independently identified as a potential mitotic regulator (Banerjee *et al.* 2007), a gene expressed by the luminal epithelium of the endometrium at the embryo implantation site (UBCi; Melner *et al.* 2006), and as a gene overexpressed in head and neck tumors (LOC92912; Seghatoleslam *et al.* 2006). The observations that cancers of several cell origins overexpress UBE2Q2 at both the transcript and protein levels (Seghatoleslam *et al.* 2006; Maeda *et al.* 2009; Nikseresht *et al.* 2010) suggested a role in promoting proliferation and/or transformation. However, it is possible that the observed overexpression is actually the indirect result of a malfunctioning feedback system. For example, expression of a cyclin E harboring mutations to confer resistance to ubiquitin-mediated proteolysis in primary fibroblasts paradoxically resulted in accumulation of the tumor suppressors p21 and p53 (Minella *et al.* 2002). Indeed, UBE2Q2 was identified on the basis of implantation-induced expression in the luminal epithelium of the endometrium at a time when the cells are undergoing differentiation and apoptosis (Melner *et al.* 2006). Similarly, a significant increase in the expression of a murine ortholog, UBE2Q1, was observed during B-cell development at a stage when proliferation is abruptly terminated (Seita *et al.* 2012). These observations correlating UBE2Q2 expression with differentiation and inhibition of proliferation are consistent with the accumulation of cells in the G_0_ and G_1_ phases upon experimental UBE2Q2 overexpression (Maeda *et al.* 2009; Seghatoleslam *et al.* 2012). Thus, it remains to be determined whether the mammalian UBE2Q2 acts similar to UBC-25 in physiologic cell-cycle quiescence.

During the course of these studies we often observed that the loss of a single gene activity did not strongly disrupt cell-cycle quiescence, whereas the combination of mutations that disrupted seemingly disconnected processes produced stronger phenotypes. These synergies illustrate the cooperation between separate activities within the regulatory network to achieve a common outcome. We expect that the highly reproducible developmental cell lineage of *C. elegans* is due in large part to the strict management conferred by the multiple processes working independently to coordinate cell-cycle entry with development. Because similar safeguards likely manage cell divisions within higher animals, further elaboration of cell-cycle quiescence regulatory networks in *C. elegans* will continue to clarify the complex mechanisms controlling cell cycles in humans.

## Supplementary Material

Supporting Information
