# Supplementary material for: A Complex Regulatory Network Coordinating Cell Cycles During C. elegans Development Is Revealed by a Genome-Wide RNAi Screen
Source: G3 (Bethesda). 2014 Feb 28;4(5):795–804. doi: 10.1534/g3.114.010546 (PMC4025478; doi:10.1534/g3.114.010546)
Supplement: Supporting Information [file supp_4_5_795__index.html]

A Complex Regulatory Network Coordinating Cell Cycles During C. elegans Development Is Revealed by a Genome-Wide RNAi Screen — Supporting Information 

# A Complex Regulatory Network Coordinating Cell Cycles During *C. elegans* Development Is Revealed by a Genome-Wide RNAi Screen

## Supporting Information for Roy *et al.*, 2014

**Files in this Data Supplement:**

- Supporting Information - Figures S1-S2 and Tables S1-S6 (PDF, 315 KB)
- Figure S1 - The VW22 strain incorporates several favorable characteristics. (PDF, 193 KB)
- Figure S2 - UBC-25 yeast two-hybrid screen identifies C30H7.2. (PDF, 178 KB)
- Table S2 - *ubc-25(ok1732)* causes temperature-sensitive viability defect. (PDF, 156 KB)
- Table S3 - The *ubc-25(ok1732)* mutation does not disturb the cell-cycle quiescence of the M, V, and Z cell lineages. (PDF, 156 KB)
- Table S4 - Comparison of wild type and *ubc-25(ok1732)* E lineage cell cycle lengths. (PDF, 151 KB)
- Table S5 - Several *ubc* genes act redundant to *ubc-25*. (PDF, 156 KB)
- Table S1 - Genes identified in the Elm phenotype RNAi screen (.xlsx, 19 KB)
- Table S6 - Examination of putative *elm* genes for enhancement of extra intestinal nuclei phenotypes (.xlsb, 56 KB)
