# Supplementary material for: A Complex Regulatory Network Coordinating Cell Cycles During C. elegans Development Is Revealed by a Genome-Wide RNAi Screen
Source: G3 (Bethesda). 2014 Feb 28;4(5):795–804. doi: 10.1534/g3.114.010546 (PMC4025478; doi:10.1534/g3.114.010546)
Supplement: Supporting Information [file supp_g3.114.010546_010546SI.pdf]

G3: Genes | Genomes | Genetics

A complex regulatory network coordinating cell cycles during *Caenorhabditis elegans* development is revealed by a genome-wide RNAi screen

Sarah H. Roy,*[1] David V. Tobin,*[1] Nadin Memar,[§] Eleanor Beltz,* Jenna Holmen,* Joseph E. Clayton,* Daniel J. Chiu,* Laura D. Young,* Travis H. Green,* Isabella Lubin,* Yuying Liu,* Barbara Conradt[§] and R. Mako Saito*,[†,2]

Author Affiliations
*Department of Genetics
Geisel School of Medicine at Dartmouth
Hanover, New Hampshire 03755 USA
[§]Center for Integrated Protein Science Munich (CiPSM)
Biocenter, LMU Munich
82152 Planegg-Martinsried, Germany
[†]Norris Cotton Cancer Center
Lebanon, New Hampshire 03755 USA

[1] These authors contributed equally to this work.
[2] Corresponding author: Dept. of Genetics and Norris Cotton Cancer Center, 601 Vail, HB 7400, Geisel School of Medicine at Dartmouth, Hanover, NH 03755. Email: Richard.M.Saito@Dartmouth.edu

DOI: 10.1534/g3.114.010546

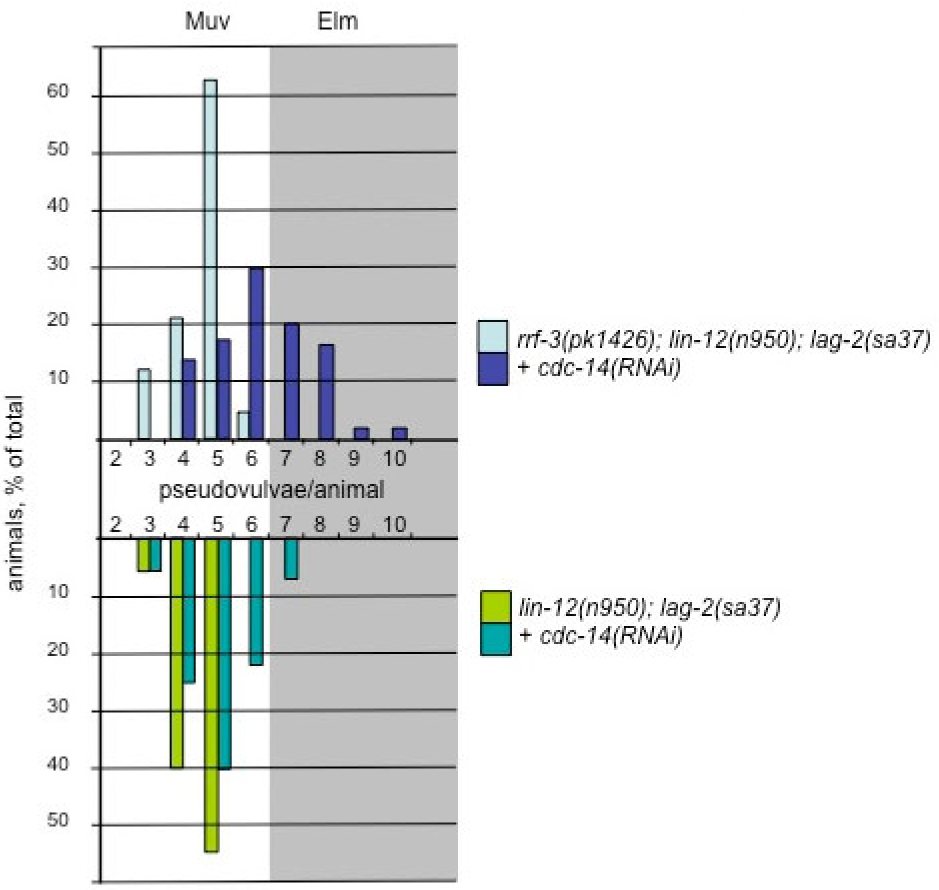

Figure S1 The VW22 strain incorporates several favorable characteristics. The *rrf-3(pk1426)* mutation enhances the Elm phenotype of *cdc-14(RNAi).* Comparison of pseudovulva number produced by *lin-12(n950); lag-2(sa37)* double mutant (lower graph) and *rrf-3(pk1426); lin-12(n950); lag-2(sa37)* triple mutant (upper graph) animals. Animals displaying the Elm phenotype are indicated by grey shading.

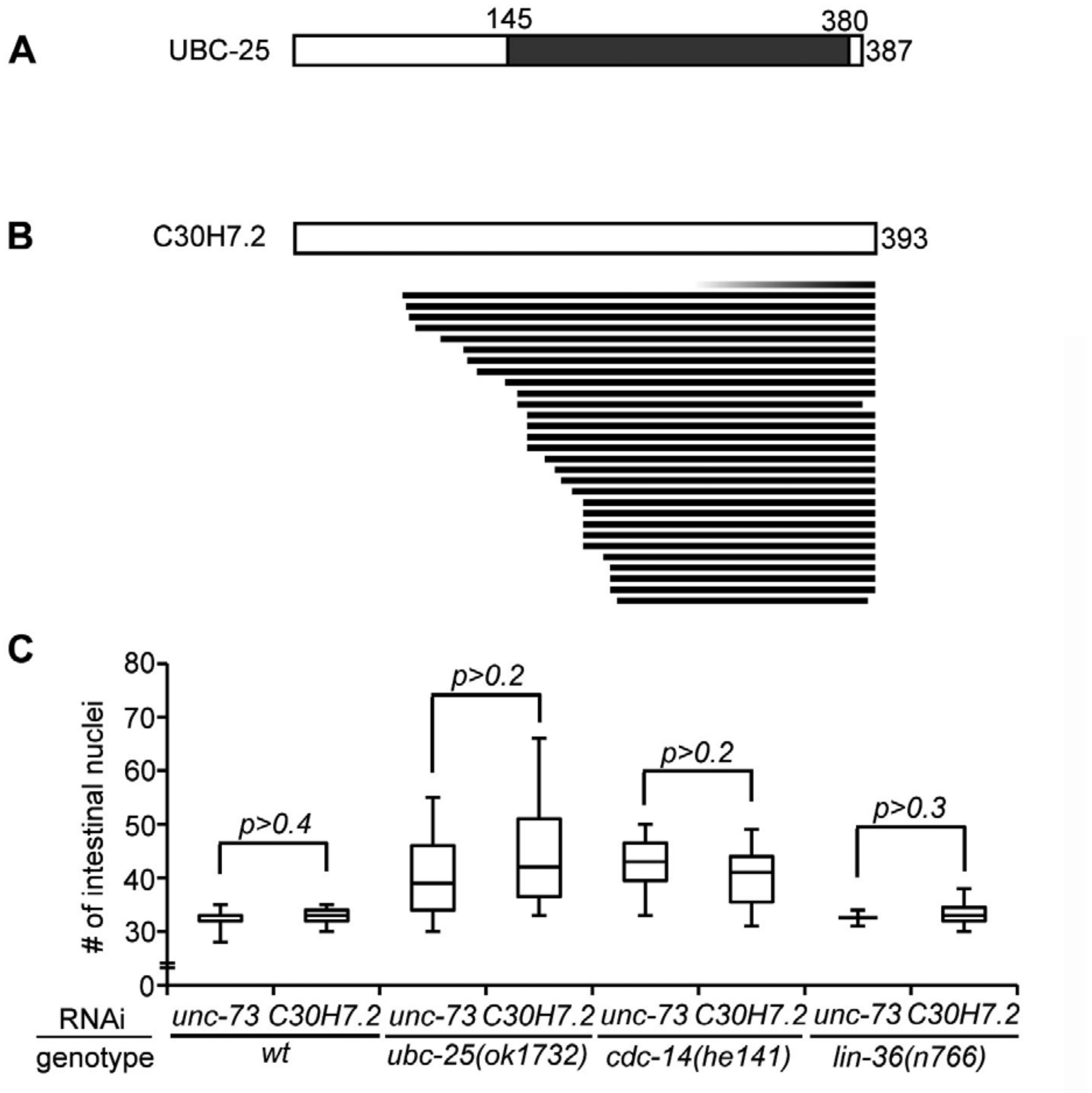

Figure S2 UBC-25 yeast two-hybrid screen identifies C30H7.2. (A) Diagram of UBC-25 open reading frame used in Y2H screen. Ubiquitin conjugating domain is shaded dark grey. The UBC-25 cDNA was cloned in-frame with the LexA DNA binding domain. Approximately $7.8x10^7$ potential interactions within a high complexity *C. elegans* cDNA library were screened. (B) Schematic diagram illustrating the open reading frames of the thirty clones (black lines) representing C30H6.2 that were isolated in the UBC-25 Y2H screen. The cDNA inserts were sequenced from both the 5' and 3' directions. The 5' sequence of a single clone was not determined and is shown as a fading black line. (C) RNAi-mediated inhibition of C30H7.2 did not significantly alter the number of intestinal nuclei in *wt*, *ubc-25(ok1732)*, *cdc-14(he141)*, or *lin-36(n766)* mutant animals. Intestinal nuclei were examined during the L4 stage.

Table S1 Genes identified in the Elm phenotype RNAi screen

Available for download as an Excel file at http://www.g3journal.org/lookup/suppl/doi:10.1534/g3.114.010546/-/DC1

Table S2 ***ubc-25(ok1732)*** **causes temperature-sensitive viability defect**

	wild type		*ubc-25(ok1732)*	
Temp.	Brood size	% Emb	Brood size	% Emb
15°C	189.4±19.7 (n=8)	0.8 (n=1510)	181.9±34.5 (n=11)	2.6 (n=2001)
20°C	273.0±34.6 (n=10)	2.7 (n=2734)	236.4±53.8 (n=11)	6.7 (n=2600)
25°C	190.6±32.5 (n=7)	1.6 (n=1576)	64.9±15.5 (n=9)	69.8 (n=584)

Table S3 The *ubc-25(ok1732)* mutation does not disturb the cell-cycle quiescence of the M, V, and Z cell lineages

lineage	age	reporter	wild type	*ubc-25(ok1732)*
M	L2	*hlh-8::GFP*	16.5±0.7 (n=24)	17.7±0.6 (n=20)
V	L4	*scm::GFP*	16.1±0.2 (n=20)*	16.1±0.6 (n=20)*
Z	L4	*lag-2::GFP*	2.0±0.0 (n=39)	2.0±0.0 (n=35)

*one side of GFP expressing V cells were counted per animal

Table S4 Comparison of wild type and *ubc-25(ok1732)* E lineage cell cycle lengths

	average cell division length*	
	wild type	*ubc-25(ok1732)*(% of wt)
cell	n=2	n=5
E	36.5±0.7	32.4±2.4 (89)
Ea	41.0±1.4	35.4±4.4 (86)
Ear	67.0±9.9	47.2±2.9 (70)
Eara	125±21.2	56.6±6.3 (45)

***time (minutes) from mitosis producing named cell to division of cell**

Table S5 Several *ubc* genes act redundant to *ubc-25*.

RNAi target*	intestinal nuclei, avg ± std dev	
	wild type	*ubc-25(ok1732)*
unc-73	33.1±1.4	38.3±6.5
cdc-14	35.3±2.8	48.5±10.2
ubc-1	33.0 ±2.6	54.5±9.2
ubc-2	Let	Let
ubc-3	31.9±1.3	38.9±5.7
ubc-6	32.6±1.2	44.5±11.3
ubc-7	32.5±1.1	36.3±7.9
ubc-8	N/A	N/A
ubc-9	34.5±3.4	Let
ubc-12	31.7±1.8	Let
ubc-13	N/A	N/A
ubc-14	N/A	N/A
ubc-15	32.5±1.1	38.8±5.0
ubc-16	32±2.0	40.6±8.3
ubc-17	32.9±1.6	50.4±12.2
ubc-18	33.2±2.6	38.2±5.6
ubc-19	N/A	N/A
ubc-20	33.3±2.4	53.9±9.3
ubc-21	31.7±0.9	47.8±13.8
ubc-22	32.6±2.1	42.7±8.9
ubc-23	32.9±1.1	45.6±7.6
ubc-24	32.6±2.0	39.1±7.7
ubc-25	32.3±2.0	44.2±10.3
ubc-26	N/A	N/A

*no *ubc*s numbered 4, 5, 10 and 11 in *C. elegans*
N/A=not available in RNAi Library
unc-73 and *cdc-14* are negative and positive controls, respectively.
n≥15 for all experiments.

Table S6 Examination of putative *elm* genes for enhancement of extra intestinal nuclei phenotypes

Available for download at http://www.g3journal.org/lookup/suppl/doi:10.1534/g3.114.010546/-/DC1.
