# Supplementary material for: A Complex Regulatory Network Coordinating Cell Cycles During C. elegans Development Is Revealed by a Genome-Wide RNAi Screen
Source: G3 (Bethesda). 2014 Feb 28;4(5):795–804. doi: 10.1534/g3.114.010546 (PMC4025478; doi:10.1534/g3.114.010546)
Supplement: Supporting Information [file supp_g3.114.010546_FigureS1.pdf]

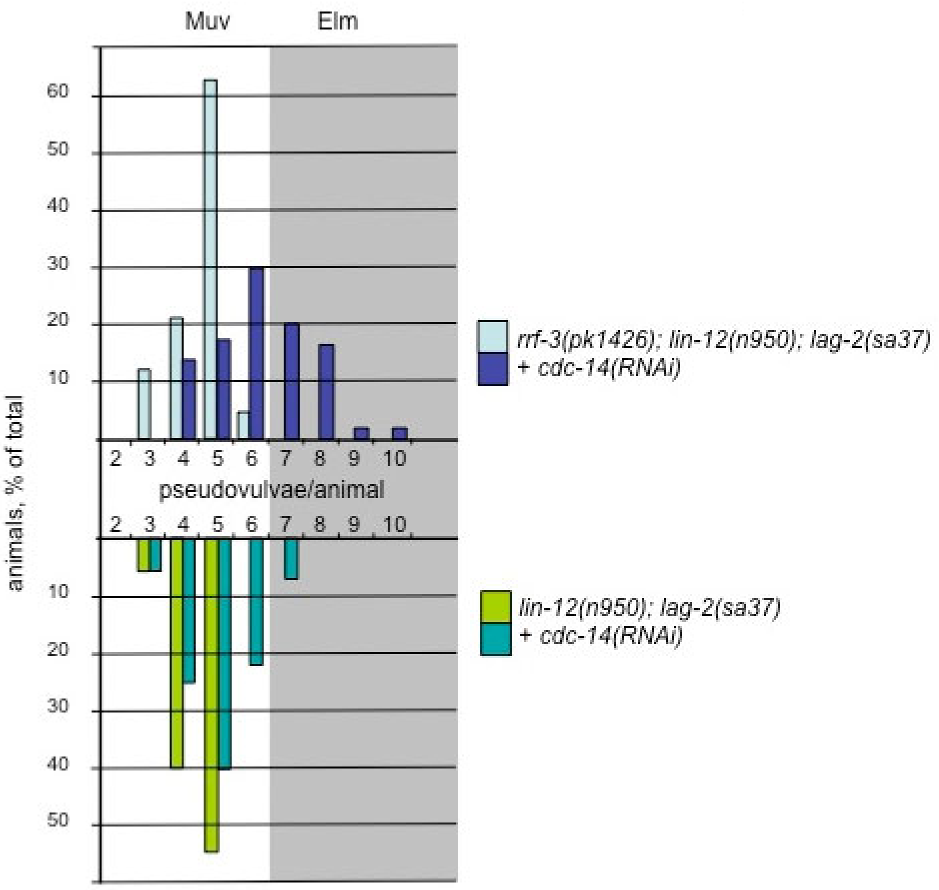

Figure S1 The VW22 strain incorporates several favorable characteristics. The *rrf-3(pk1426)* mutation enhances the Elm phenotype of *cdc-14(RNAi).* Comparison of pseudovulva number produced by *lin-12(n950); lag-2(sa37)* double mutant (lower graph) and *rrf-3(pk1426); lin-12(n950); lag-2(sa37)* triple mutant (upper graph) animals. Animals displaying the Elm phenotype are indicated by grey shading.
