# Supplementary material for: A Complex Regulatory Network Coordinating Cell Cycles During C. elegans Development Is Revealed by a Genome-Wide RNAi Screen
Source: G3 (Bethesda). 2014 Feb 28;4(5):795–804. doi: 10.1534/g3.114.010546 (PMC4025478; doi:10.1534/g3.114.010546)
Supplement: Supporting Information [file supp_g3.114.010546_FigureS2.pdf]

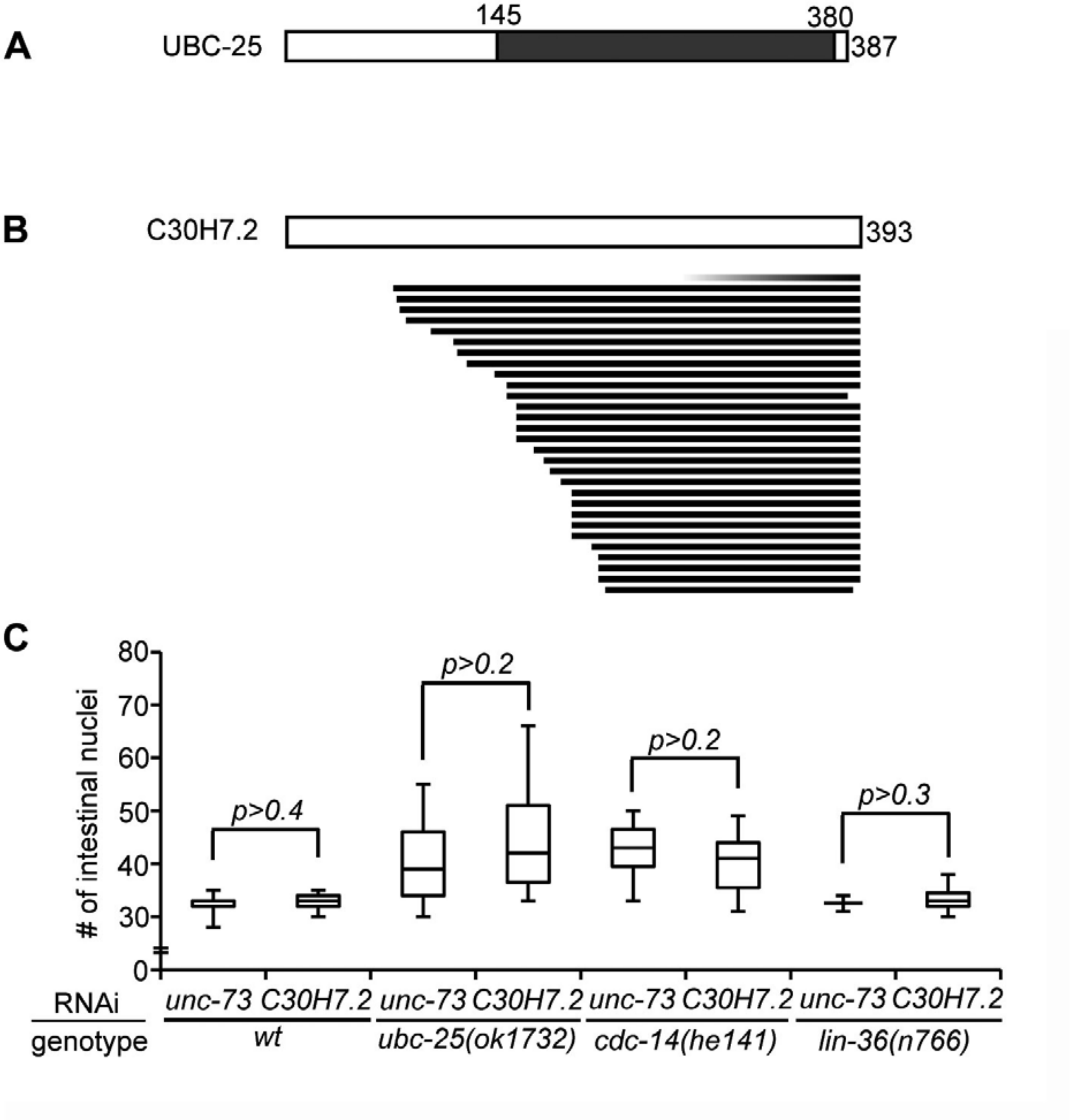

Figure S2 UBC-25 yeast two-hybrid screen identifies C30H7.2. (A) Diagram of UBC-25 open reading frame used in Y2H screen. Ubiquitin conjugating domain is shaded dark grey. The UBC-25 cDNA was cloned in-frame with the LexA DNA binding domain. Approximately $7.8x10^7$ potential interactions within a high complexity *C. elegans* cDNA library were screened. (B) Schematic diagram illustrating the open reading frames of the thirty clones (black lines) representing C30H6.2 that were isolated in the UBC-25 Y2H screen. The cDNA inserts were sequenced from both the 5' and 3' directions. The 5' sequence of a single clone was not determined and is shown as a fading black line. (C) RNAi-mediated inhibition of C30H7.2 did not significantly alter the number of intestinal nuclei in *wt*, *ubc-25(ok1732)*, *cdc-14(he141)*, or *lin-36(n766)* mutant animals. Intestinal nuclei were examined during the L4 stage.
