# Supplementary material for: A Complex Regulatory Network Coordinating Cell Cycles During C. elegans Development Is Revealed by a Genome-Wide RNAi Screen
Source: G3 (Bethesda). 2014 Feb 28;4(5):795–804. doi: 10.1534/g3.114.010546 (PMC4025478; doi:10.1534/g3.114.010546)
Supplement: Supporting Information [file supp_g3.114.010546_TableS2.pdf]

Table S2 ***ubc-25(ok1732)*** **causes temperature-sensitive viability defect**

	wild type		*ubc-25(ok1732)*	
Temp.	Brood size	% Emb	Brood size	% Emb
15°C	189.4±19.7 (n=8)	0.8 (n=1510)	181.9±34.5 (n=11)	2.6 (n=2001)
20°C	273.0±34.6 (n=10)	2.7 (n=2734)	236.4±53.8 (n=11)	6.7 (n=2600)
25°C	190.6±32.5 (n=7)	1.6 (n=1576)	64.9±15.5 (n=9)	69.8 (n=584)
