# Supplementary material for: A Complex Regulatory Network Coordinating Cell Cycles During C. elegans Development Is Revealed by a Genome-Wide RNAi Screen
Source: G3 (Bethesda). 2014 Feb 28;4(5):795–804. doi: 10.1534/g3.114.010546 (PMC4025478; doi:10.1534/g3.114.010546)
Supplement: Supporting Information [file supp_g3.114.010546_TableS3.pdf]

Table S3 The *ubc-25(ok1732)* mutation does not disturb the cell-cycle quiescence of the M, V, and Z cell lineages

lineage	age	reporter	wild type	*ubc-25(ok1732)*
M	L2	*hlh-8::GFP*	16.5±0.7 (n=24)	17.7±0.6 (n=20)
V	L4	*scm::GFP*	16.1±0.2 (n=20)*	16.1±0.6 (n=20)*
Z	L4	*lag-2::GFP*	2.0±0.0 (n=39)	2.0±0.0 (n=35)

*one side of GFP expressing V cells were counted per animal
