# Supplementary material for: A Complex Regulatory Network Coordinating Cell Cycles During C. elegans Development Is Revealed by a Genome-Wide RNAi Screen
Source: G3 (Bethesda). 2014 Feb 28;4(5):795–804. doi: 10.1534/g3.114.010546 (PMC4025478; doi:10.1534/g3.114.010546)
Supplement: Supporting Information [file supp_g3.114.010546_TableS4.pdf]

Table S4 Comparison of wild type and *ubc-25(ok1732)* E lineage cell cycle lengths

	average cell division length*	
	wild type	*ubc-25(ok1732)*(% of wt)
cell	n=2	n=5
E	36.5±0.7	32.4±2.4 (89)
Ea	41.0±1.4	35.4±4.4 (86)
Ear	67.0±9.9	47.2±2.9 (70)
Eara	125±21.2	56.6±6.3 (45)

***time (minutes) from mitosis producing named cell to division of cell**
