# Supplementary material for: A Complex Regulatory Network Coordinating Cell Cycles During C. elegans Development Is Revealed by a Genome-Wide RNAi Screen
Source: G3 (Bethesda). 2014 Feb 28;4(5):795–804. doi: 10.1534/g3.114.010546 (PMC4025478; doi:10.1534/g3.114.010546)
Supplement: Supporting Information [file supp_g3.114.010546_TableS5.pdf]

Table S5 Several *ubc* genes act redundant to *ubc-25*.

	intestinal nuclei, avg ± std dev	
RNAi target*	wild type	*ubc-25(ok1732)*
unc-73	33.1±1.4	38.3±6.5
cdc-14	35.3±2.8	48.5±10.2
ubc-1	33.0 ±2.6	54.5±9.2
ubc-2	Let	Let
ubc-3	31.9±1.3	38.9±5.7
ubc-6	32.6±1.2	44.5±11.3
ubc-7	32.5±1.1	36.3±7.9
ubc-8	N/A	N/A
ubc-9	34.5±3.4	Let
ubc-12	31.7±1.8	Let
ubc-13	N/A	N/A
ubc-14	N/A	N/A
ubc-15	32.5±1.1	38.8±5.0
ubc-16	32±2.0	40.6±8.3
ubc-17	32.9±1.6	50.4±12.2
ubc-18	33.2±2.6	38.2±5.6
ubc-19	N/A	N/A
ubc-20	33.3±2.4	53.9±9.3
ubc-21	31.7±0.9	47.8±13.8
ubc-22	32.6±2.1	42.7±8.9
ubc-23	32.9±1.1	45.6±7.6
ubc-24	32.6±2.0	39.1±7.7
ubc-25	32.3±2.0	44.2±10.3
ubc-26	N/A	N/A

*no *ubc*s numbered 4, 5, 10 and 11 in *C. elegans*
N/A=not available in RNAi Library
unc-73 and *cdc-14* are negative and positive controls, respectively.
n≥15 for all experiments.
